# The Validation of Deep Learning-Based Grading Model for Diabetic Retinopathy

**DOI:** 10.3389/fmed.2022.839088

**Published:** 2022-05-16

**Authors:** Wen-fei Zhang, Dong-hong Li, Qi-jie Wei, Da-yong Ding, Li-hui Meng, Yue-lin Wang, Xin-yu Zhao, You-xin Chen

**Affiliations:** ^1^Department of Ophthalmology, Peking Union Medical College Hospital, Chinese Academy of Medical Sciences, Beijing, China; ^2^Key Laboratory of Ocular Fundus Diseases, Chinese Academy of Medical Sciences & Peking Union Medical College, Beijing, China; ^3^Visionary Intelligence Ltd., Beijing, China

**Keywords:** diabetic retinopathy, artificial intelligence, validation, eye wisdom V1, sensitivity, specificity

## Abstract

**Purpose:**

To evaluate the performance of a deep learning (DL)-based artificial intelligence (AI) hierarchical diagnosis software, EyeWisdom V1 for diabetic retinopathy (DR).

**Materials and Methods:**

The prospective study was a multicenter, double-blind, and self-controlled clinical trial. Non-dilated posterior pole fundus images were evaluated by ophthalmologists and EyeWisdom V1, respectively. The diagnosis of manual grading was considered as the gold standard. Primary evaluation index (sensitivity and specificity) and secondary evaluation index like positive predictive values (PPV), negative predictive values (NPV), etc., were calculated to evaluate the performance of EyeWisdom V1.

**Results:**

A total of 1,089 fundus images from 630 patients were included, with a mean age of (56.52 ± 11.13) years. For any DR, the sensitivity, specificity, PPV, and NPV were 98.23% (95% CI 96.93–99.08%), 74.45% (95% CI 69.95-78.60%), 86.38% (95% CI 83.76-88.72%), and 96.23% (95% CI 93.50-98.04%), respectively; For sight-threatening DR (STDR, severe non-proliferative DR or worse), the above indicators were 80.47% (95% CI 75.07-85.14%), 97.96% (95% CI 96.75-98.81%), 92.38% (95% CI 88.07-95.50%), and 94.23% (95% CI 92.46-95.68%); For referral DR (moderate non-proliferative DR or worse), the sensitivity and specificity were 92.96% (95% CI 90.66-94.84%) and 93.32% (95% CI 90.65-95.42%), with the PPV of 94.93% (95% CI 92.89-96.53%) and the NPV of 90.78% (95% CI 87.81-93.22%). The kappa score of EyeWisdom V1 was 0.860 (0.827-0.890) with the AUC of 0.958 for referral DR.

**Conclusion:**

The EyeWisdom V1 could provide reliable DR grading and referral recommendation based on the fundus images of diabetics.

## Introduction

Diabetic retinopathy (DR) is one of the leading causes of blindness among working-age people, about one-third of the diabetic population have clinically visible DR, another one-third might suffer a sight-threatening state of the disease, characterized as proliferative DR (PDR) or diabetic macular edema (DME) (1). There is considerable heterogeneity in the incidence of DR around the world and within countries (2). In China, the prevalence of DR in diabetic patients is 24.7-37.0%, the proportion of referable DR (moderate non-proliferative DR or worse) is around 25% and sight-threatening DR (STDR, severe non-proliferative DR or worse) is about 11%, which were reported to increase rapidly (3–7).

As the early stage of DR is usually asymptomatic, only 36.5% of diabetic patients in China are aware of this disease, even fewer patients could get timely interventions (8). Numerous DR patients might progress to a late-stage and suffer severe and irreversible visual loss. Thus, early detection and timely referral are of great value in delaying the progress of DR and preventing vision loss. Although advanced fundus cameras and telehealth services were widespread in China, the workload of DR screening was too overwhelming, and the number of experienced ophthalmologists was also scarce. Meanwhile, several studies have shown that certified ophthalmologists screening DR by indirect ophthalmoscopy could only achieve an average sensitivity of 33, 34, or 73% (9–12). The huge number of people with diabetes, the shortage of ophthalmologists, and the imbalance of medical resources all present as major obstacles to early detection and timely intervention for DR. Therefore, there is an urgent need to establish a sound monitoring and follow-up strategy for diabetic patients.

Deep learning (DL), as a subfield of artificial intelligence (AI), has shown a convincing performance in the diagnosis of diabetic retinopathy by fundus images and peripheral neuropathy in diabetes mellitus utilizing corneal confocal microscopy (CCM). DL-based technology has been applied to retinal vascular segmentation, recognition, and classification of DR lesions, referable DR and diabetic neuropathy detection. Recently, Preston et al. developed an AI-based algorithm to classify peripheral neuropathy utilizing CCM without image segmentation prior to classification, which did not require manual or automated annotation and allowed the utilization of a larger database (13). Most of the above algorithms use the convolutional neural networks (CNN) architecture, which has better performance than other network architectures (14). At the same time, DL has the advantages of continuous work, no need to rest, and reproducibility, so it does not need to spend a lot of manpower to train doctors. In addition, the application of DL in DR referral is conducive to the discovery of DR patients in remote and poor areas and has the potential to reduce ophthalmologists’ workload and improve the efficiency of DR screening programs (15).

Although several studies have achieved high sensitivity and specificity of DL in the recognition of DR, some controversies and defects still exist (16–20). The distribution of the data sets like Eyepacs-1 test and Messidor-2 used in previous studies were extremely unbalanced, with negative data accounting for more than 90% and mainly concentrating on grade 0. As Chinese hospitals have a much higher proportion of positive data, the sensitivity and specificity of diagnostic tests will be affected (4, 7). Thus, we designed this prospective multicenter clinical trial to evaluate the performance and feasibility of a CNN-based AI software we invented, EyeWisdom V1 (Visionary Intelligence Ltd., Beijing, China), which could offer 5-stage DR diagnosis and referable recommendation.

## Materials and Methods

This prospective clinical trial was a multicenter (Peking Union Medical College Hospital, Eye Hospital China Academy of Chinese Medical Sciences, and Beijing Friendship Hospital of Capital Medical University), double-masked and self-control trial, aiming to evaluate the feasibility and safety of EyeWisdom V1 for the diagnosis of DR through the comparison with manual grading. All patients signed an informed consent form (ICF) approved by the Ethics Committee (EC) (or exempted from signing the ICF authorized by the Ethics Committee). This clinical trial met the ICMJE requirements and was registered in the Beijing food and drug administration of China (No.20180294).

**FIGURE 1:**
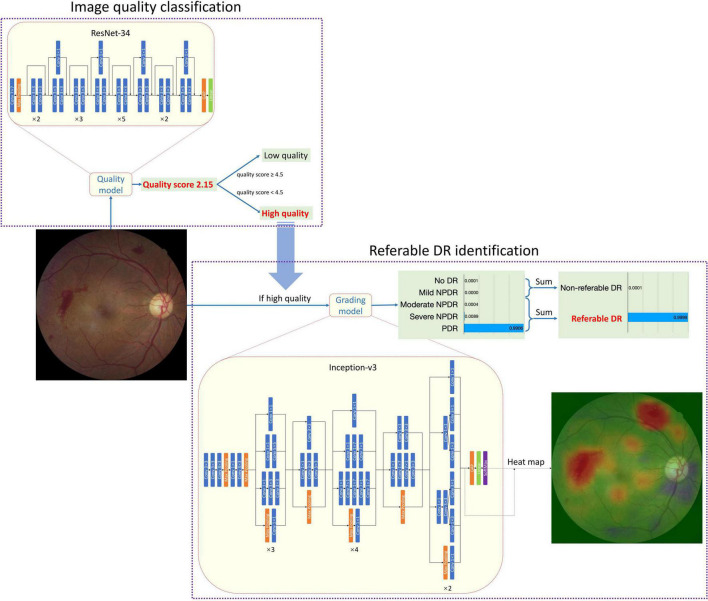
The network architecture of EyeWisdom V1.

Figure 1 shows the architecture of the whole AI Software and the individual architecture of the quality control (QC) module and DR classification module. Resnet-34 and Inception-V3 network was used in the QC module and DR classification module, respectively. ResNet was a CNN architecture proposed by He et al. (21). It consisted of special residual blocks which had skip connections from the start to the end. Therefore, the output of each residual block was calculated as the sum of the original input and the results of convolutions. This design could decrease the optimization complexity so that better performances could be achieved when networks went deeper. ResNet-34 had 34 trainable layers (convolutional layer and fully connected layer) in total. We replaced the output neuron number of the last fully connected layer from 1,000 to 1 for quality control. Inception-V3 was proposed by Szegedy et al. (22). It had different inception blocks. Each block had branches that consisted of convolutional layers with different kernel sizes. Therefore, the blocks could capture features of multiple sizes. Considering that the size of DR-related lesions ranged from very small microaneurysm to large vitreous hemorrhages, the characteristic of Inception-V3 was crucial. The output neuron number of the last fully connected layer was replaced from 1,000 to 5 corresponding to 5 level DR grades. Images would first go through the QC module and only those judged as “gradable” would be passed to the DR classification module. Both the QC module and DR classification module were trained with Stochastic Gradient Descent (SGD). The momentum was set to 0.9 and the weight decay was set to 0.0001. The initial learning rate was 0.001. The learning rate was divided by 10 if the performance had not increased in 4 consistent validations. The training stopped if the performance had not increased in 10 consistent validations. Considering the risk of overfitting and the imbalance of data distribution, data augmentation was adopted during training. The QC module was trained with Mean Squared Error Loss and the DR classification module was trained with Kappa Loss.

The development of the software, including the QC module and DR classification module, and the performance of the AI Software in the internal test set was presented in Supplementary Datasheet 1. To find out the best network for DR classification, we compared the different combinations of three mature network architectures (VGG16, ResNet-50, and Inception-V3) and image size. (Supplementary Table 1 and Supplementary Figure 1 shows the comparison of different combinations of network and image size. Supplementary Tables 2, 3 show the data distribution of different sets in two modules.). After comparison, the Inception-V3 network, with an input image size of 896*896 was selected. Since the CNN was a black-box algorithm, we adopted a heat map for a better understanding of the CNN output. The activated areas in the corresponding heat map showed why the image was predicted to a specific class. The example in Figure 1 showed that the main reason that the fundus image was predicted as PDR was the two pre-retinal hemorrhages, which was similar to human doctors’ logic. The Inception-V3 network took 896*896 fundus images as input and output five scores at the image level. There was no human–AI interaction in the handling of the input data. The five scores represented the network’s certainty of no DR, mild NPDR, moderate NPDR, serve NPDR, and PDR (or DR0 to DR4 for short), respectively.

### Fundus Photography Acquisition

The fundus cameras used in this study included Topcon TRC-NW6S, Cannon CR2, and KOWA Nonmyd α-DIII 8300 from 3 centers (Peking Union Medical College Hospital, Eye Hospital China Academy of Chinese Medical Sciences and Beijing Friendship Hospital of Capital Medical University). Non-dilated, the posterior pole images containing the optic disk and macula were included. To ensure the recognition effect the images should be at least 30 pixels/degree. The horizontal and vertical field of view should be at least 45° and 40°. The image compression ratio should not be greater than 25. The literature has demonstrated that the accuracy of using this 1 image to judge grading and screening is close to that of 7 images (23, 24).

### Image Quality Assessment

Before the inclusion of fundus images, evaluation of image quality was conducted through manual screening by ophthalmologists. Image quality assessment criteria were based on the following factors: (1) focus; (2) brightness; (3) the area of the image includes the entire optic disc and macula or not; and (4) there are defects in the image that affect the classification judgment or not (e.g., dust spots, deformation, eyelashes). Image quality was classified as “excellent,” which means all lesions can be graded, “good” if there are only 1–2 factors affecting image quality, “adequate quality” if there are 3–4 problems that affect image quality but all lesions grading is not affected, “insufficient for full interpretation” if one or more lesions cannot be graded, “insufficient for any interpretation” and “others” if some other quality factors interfered with grading.

### Inclusion and Exclusion

Diabetic patients and their fundus images for inclusion in this study must meet all the following criteria.

(1) Images of patients who have signed the ICF or who have been authorized by the ethics committee to refrain from signing the informed consent.
(2) Patients with confirmed diabetes mellitus.
(3) Information about the patients, including name, age, sex and time of screening, was available.
(4) Fundus images were gradable and should contain the optic disk and macula.

Participants and their fundus images would not be included if they meet any of the following criteria.

(1) Patients with gestational diabetes mellitus.
(2) Patients that received the laser and surgical treatment of eyes.
(3) Patients with other retinal vascular diseases, such as retinal vein occlusion and retinal vasculitis.
(4) Patients with incomplete information related to the disease.
(5) Images failed to meet the gradable requirements.

### Manual and Software Grading

**FIGURE 2:**
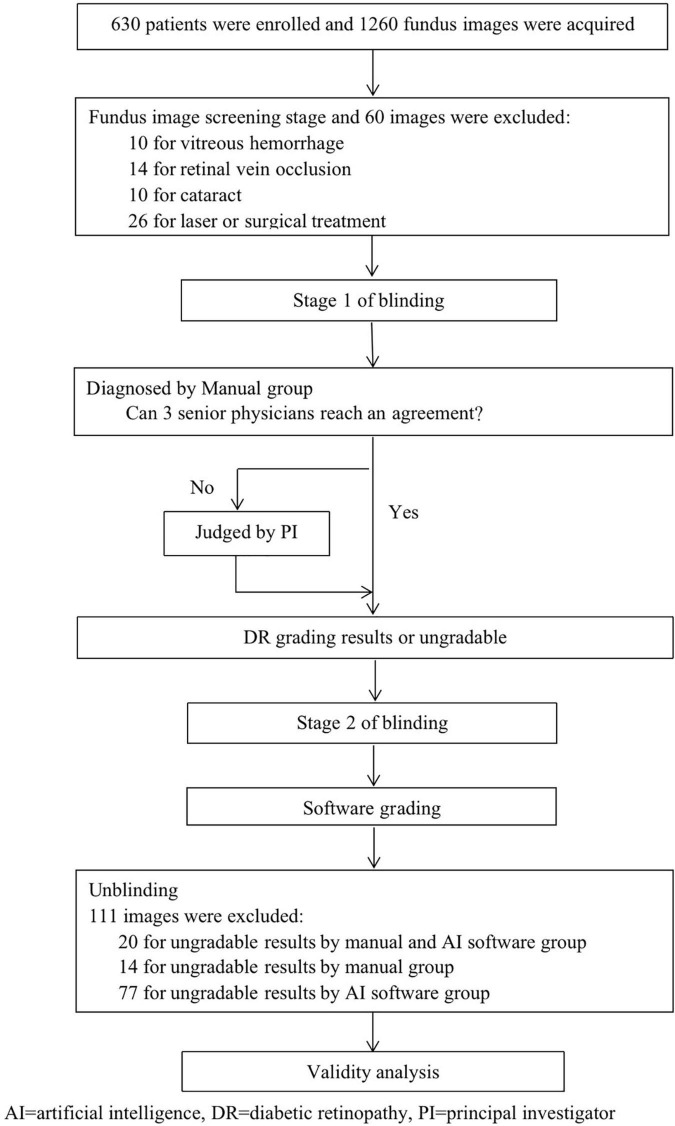
The work flowchart of fundus image grading.

In this study, a fundus image was independently diagnosed by EyeWisdom V1 and ophthalmologists (which was the gold standard). A simple flow chart of the study is shown in Figure 2. Before blinding, the included images were numbered in accordance with the order of inclusion (pre-test number). SPSS V.26. was used to randomly generate two blind tables, manual grading number (sequence 1) and software grading number (sequence 2). Stage 1 of blinding: blinding was performed by an individual independent of the study. The pre-test number was masked and converted to sequence 1. Manual grading: manual grading group consisted of three senior physicians and one principal investigator (PI). Three senior physicians were provided by each center and completed the diagnosis in the masked fashion. If the three senior physicians could reach an agreement, the grades would be included as the final diagnosis. If not, the PI’s opinion would serve as the final grade. Stage 2 of blinding: Sequence 1 was masked and converted to sequence 2. Software grading: AI identified and completed the diagnosis automatically and the software operator was the ophthalmologists of each center.

### Calculation of Sample Size

The sample size calculated in this study was determined on the basis of indicators of effectiveness. This clinical study was designed with a single-group target value method and the primary endpoint indicators were sensitivity, specificity, and diagnostic accordance rate (DAR) under the observation target items. The calculation formula was as follows.


(1) $$n=\frac{{\left[{\text{Z}}_{1-\alpha /2}\,\sqrt{P_{0}\,\left(1-P_{0}\right)}+Z_{1-\beta }\,\sqrt{P_{T}\,\left(1-P_{T}\right)}\right]}^{2}}{{\left({\text{P}}_{T}-P_{0}\right)}^{2}}$$

*n* = sample size, *P*_0_ = target values, *P*_T_ = expected values, *Z*_1–α/2_ and *Z*_1–β_ were from Z score table.

After comprehensively referring to the indicators at home and abroad and the ophthalmologists’ ability in Peking Union Medical College Hospital and Eye Hospital China Academy of Chinese Medical Sciences, we finally set the main evaluation indicators for this clinical study: the target values of sensitivity and specificity were 85% and 80%, respectively (equivalent to the diagnostic ability of doctors in lower hospitals). Meanwhile, according to the pre-test of this software (Supplementary Datasheet 2 and Supplementary Tables 4 -7 shows the details of the pre-test), the expected values of sensitivity and specificity were set as 89 and 85%, respectively.

According to the sensitivity results, target value P0 was set as 85.0% and expected value Pt was set as 89.0%, 580 positive samples were needed. According to the specificity results, the target value P0 was set as 80.0% and the expected value PT was set as 85.0%, 470 negative samples were needed. Finally, the total number of cases was 1,050. The trial was carried out in three centers and the shedding factors were controlled by 20%. Therefore, the sample size was enlarged to 1,260 images (630 subjects included). The three test centers were allocated according to the ratio of 1:1:1 and 420 images (210 subjects included) were required for each center.

### Primary and Secondary Outcome

Primary evaluation index (sensitivity and specificity), as well as secondary evaluation index such as positive predictive values (PPV), negative predictive values (NPV), the quadratic weighted kappa score and the area under the receiver operating characteristic curve (AUC) were calculated to evaluate the performance of EyeWisdom V1.

### Statistical Analysis

SPSS V.26 was used to analyze the sensitivity, specificity, negative predictive value (NPV), and positive predictive value (PPV) of the DL algorithm in detecting any DR, referable DR, and sight-threatening DR (STDR). For referable DR and any DR, the corresponding quadratic weighted kappa and DAR between EyeWisdom V1 grading and manual grading were also calculated. We also analyzed the AUC of software grading and manual grading. The 95%CI of the sensitivity, specificity, DAR, PPV, and NPV were calculated using the Clopper-Pearson method (25). *P* value less than 0.05 would be considered statistically significant.

## Results

### Baseline Characteristics

A total of 630 people and 1,089 eyes from August 2018 to July 2019 were included in this study, of which 60 fundus images were excluded mainly due to concomitant with other fundus diseases such as retinal vein occlusion or previous laser and surgical treatment, and another 111 fundus photos were excluded due to the inability to obtain the diagnostic results of ophthalmologists and software at the same time. 640 of the images were from men. The mean (SD) age of all participants was 56.52 (11.13). In these images, 551 were from the right eye.

### Image Quality Assessment

Image quality was assessed by manual screening and 994 images were classified as excellent, 94 as good, and 1 as adequate. All the 1,089 images were evaluated as gradable. Among 111 pictures that were excluded, 20 were labeled as ungradable by manual and software grading, 14 by manual grading, and 77 by software grading (Figure 2). The DAR between manual and software groups was 92.42% (95%CI 90.77-93.85%) for image quality assessment.

### Primary and Secondary Diagnostic Indicators

The distribution of DR staging between manual and software grading is shown in Table 1. For detecting any DR, the software had the 98.23% (95%CI 96.93-99.08%) sensitivity and 74.45% (95%CI 69.95-78.60%) specificity, with the PPV of 86.38% (95%CI 83.76-88.72%) and NPV of 96.23% (95%CI 93.50-98.04%), respectively. For detecting STDR, the sensitivity, specificity, PPV, and NPV were 80.47% (95%CI 75.07-85.14%), 97.96% (95%CI 96.75-98.81%), 92.38% (95%CI 88.07-95.50%) and 94.23% (95%CI 92.46-95.68%), respectively. For detecting referable DR, the sensitivity and specificity were relatively high, which were 92.96% (95%CI 90.66-94.84%) and 93.32% (95%CI 90.65-95.42%), respectively, with the 94.93% (95%CI 92.89-96.53%) PPV, and 90.78% (95%CI 87.81-93.22%) NPV (Table 2).

**TABLE 1:** Distribution of manual grading and AI software grading for DR.

AI grading	Manual grading					Total
	0	1	2	3	4	
0	306	2	9	0	1	318(29.20%)
1	84	41	34	0	0	159(14.60%)
2	21	9	310	40	9	389(35.70%)
3	0	0	15	110	14	139(12.80%)
4	0	1	1	3	79	84(7.70%)
Total	411 (37.74%)	53 (4.87%)	369 (33.88%)	153 (14.05%)	103 (9.46%)	1089 (100%)

*AI = artificial intelligence, DR = diabetic retinopathy*

**TABLE 2:** Performance of EyeWisdom V1 in comparison to manual grading.

Category	Any DR detection(95% CI)	Referable DR detection(95% CI)	STDR detection(95% CI)
Sensitivity	98.23% (96.93% ∼ 99.08%)	92.96% (90.66% ∼ 94.84%)	80.47% (75.07% ∼ 85.14%)
Specificity	74.45% (69.95% ∼ 78.60%)	93.32% (90.65% ∼ 95.42%)	97.96% (96.75% ∼ 98.81%)
PPV	86.38% (83.76% ∼ 88.72%)	94.93% (92.89% ∼ 96.53%)	92.38% (88.07% ∼ 95.50%)
NPV	96.23% (93.50% ∼ 98.04%)	90.78% (87.81% ∼ 93.22%)	94.23% (92.46% ∼ 95.68%)

*AI = artificial intelligence, CI = confidence interval, DR = diabetic retinopathy, NPV = negative predictive values, PPV = positive predictive values, STDR = sight threatening DR.*

When detecting referable DR, the corresponding quadratic weighted kappa between EyeWisdom V1 and manual grading was 0.860 (95%CI 0.827-0.890), which indicated “very good” agreement between these two groups. The DAR was 93.11% (95%CI 91.44-94.54%) (Table 3). As shown in Table 3, the DAR between every grader (A, B, C) and the gold standard, was 97.06% (95%CI 95.88-97.98%), 96.32% (95%CI 95.03-97.36%) and 92.47% (95%CI 90.74-93.97%), respectively, for the referable DR. When considering the kappa between every grader and reference standard, they were 0.940 (95%CI 0.927-0.953), 0.929 (95%CI 0.915-0.943), and 0.844 (95%CI 0.823-0.865). Table 3 also shows the DAR and kappa among AI software, every grader (A, B, C), and the gold standard for detecting any DR. The DAR and kappa among graders for detecting any DR and referable DR are shown in Supplementary Datasheet 3.

**TABLE 3:** The DAR and kappa between AI software, every grader (A,B,C) and gold standard for detecting any DR and referable DR.

	Any DR detection(95% CI)		Referable DR detection(95% CI)	
	DAR	Kappa	DAR	Kappa
Grader A	92.19% (90.44% ∼ 93.72%)	0.930 (0.915 ∼ 0.944)	97.06% (95.88% ∼ 97.98%)	0.940 (0.927 ∼ 0.953)
Grader B	88.61% (86.58% ∼ 90.44%)	0.927 (0.912 ∼ 0.942)	96.32% (95.03% ∼ 97.36%)	0.929 (0.915 ∼ 0.943)
Grader C	80.53% (78.05% ∼ 82.85%)	0.804 (0.779 ∼ 0.828)	92.47% (90.74% ∼ 93.97%)	0.844 (0.823 ∼ 0.865)
AI group	89.30% (87.30% ∼ 91.00%)	0.761 (0.734 ∼ 0.787)	93.11% (91.44% ∼ 94.54%)	0.860 (0.827 ∼ 0.890)

*AI = artificial intelligence, CI = confidence interval, DAR = diagnostic accordance rate, DR = diabetic retinopathy.*

**FIGURE 3:**
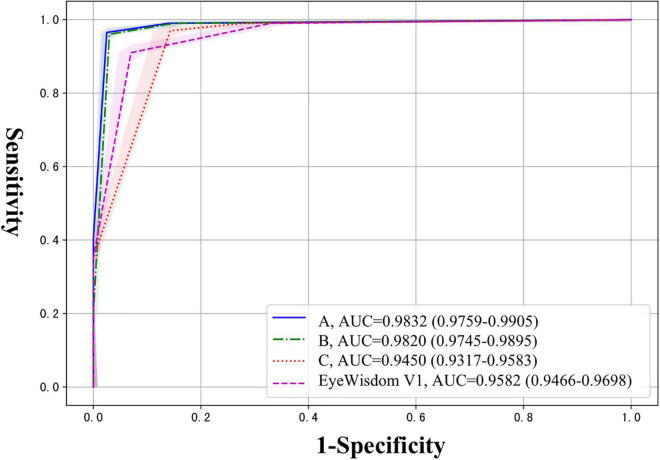
The AUC of three graders and EyeWisdom V1 for referable DR.

At the same time, we calculated the AUC for referable DR, which reflected the diagnostic effectiveness of different graders and software. As shown in Figure 3, the AUC of the three graders were 0.983 (95%CI 0.9759-0.9905), 0.982 (95%CI 0.9745-0.9895), and 0.945 (95%CI 0.9317-0.9583) respectively, and the AUC of EyeWisdom V1 was 0.958 (95%CI 0.9466-0.9698). We also compared the diagnostic results of different fundus cameras with the gold standard and the results showed that the differences were not statistically significant (*p* = 0.147) (Table 4).

**TABLE 4:** Diagnostic results of different fundus cameras.

Category	Topcon TRC-NW6S	Cannon CR2	KOWA Nonmyd α -DIII 8300
Consistent with the gold standard	219(73.7)	353(79.7)	274(78.5)
Inconsistent with the gold standard	78(26.3)	90(37.0)	75(21.5)
Total	297(100)	443(100)	349(100)

We also evaluated the safety of EyeWisdom V1 in this clinical trial. As the objects of the study were fundus images and the results were not used as the basis for any diagnosis or treatment, adverse events and serious adverse events were not defined in this clinical trial. The safety of the software was mainly to evaluate the operation failures and device defects in the application process. In this clinical trial, a total of 0 cases of device defects and operational failures were reported during the trial and the incidence was 0%.

## Discussion

Although many studies have reported the application of AI in referral DR, their performance and reliability might vary due to differences in graders, disease distribution, and reference standards (17). This prospective, multi-center, double-blind, and self-controlled clinical trial demonstrated the efficacy of an AI software, EyeWisdom V1, in the diagnosis of DR by comparing it with manual grading. Based on the image recognition technology of DL, this AI software firstly judged whether the quality of fundus images was acceptable. If the quality was acceptable, the grading results would be given based on fundus images and the recommendation of referral/non-referral would be provided at the same time. Compared with the manual grading by experience ophthalmologists (gold standard), this study confirmed that EyeWisdom V1 had acceptable sensitivity, specificity, and AUC, it also showed good strength of agreement in DR severity grading.

For the image quality assessment, our results showed a DAR of 92.24% (95%CI 90.77-93.85%) with the gold standard. After manually excluding images with other co-morbidity as well as images after laser and surgical treatment, the number of “undiagnosable results” of software grading was 77, compared with 14 of manual grading. The reason for the “undiagnosable results” of software was inadequate image quality. From the perspective of the overall images, the software had a high consistency in the judgment of image quality with the gold standard. In order to serve the screening scene, the QC module adopted a more conservative design, which was more sensitive, thus the number of images with insufficient quality diagnosed by EyeWisdom V1 was slightly more than that by manual grading.

For referral DR, the sensitivity and specificity were 92.96% (90.66-94.84%) and 93.32% (90.65-95.42%), with the PPV of 94.93% (92.89-96.53%), NPV of 90.78% (87.81-93.22%), and kappa of 0.860 (0.827-0.890), which reached the single target values set in the pre-text. At the same time, the AUC of AI software diagnosis was 0.95, which proved the acceptable diagnostic efficiency.

Cameras used in three centers were Topcon TRC-NW6S, Cannon CR2, and Kowa NonmyDα-DIII 8300, respectively. Our results showed that there was no statistically significant difference between the AI results of each center and the gold standard, no matter for any DR or referral DR. In actual use, differences in image diagnosis may come from the diversity of devices. Our results proved that the EyeWisdom V1 had good stability and robustness for different cameras.

There were many DL models using other algorithms and research methods, and the performance of these studies varied from each other. Compared to the previous clinical trials currently known to us, the pivotal trial of IDX-DR, for example, was conducted at the patient level and delivered the results of the worse eye. While our study was conducted at the image level. Their IDX-DR was bound to a Topcon NW400 camera, while we did analyze the effects of different cameras in our clinical trial (10). Most deep learning models concentrated on referral/non-referral DR based on public databases (17, 20, 26–28). While EyeWisdom V1 provided DR grading scores and classification of DR. Databases came from Chinese public hospitals, which could offer more details and more valuable references. Table 5 shows several state-of-the-art DL models for DR classification in detail.

**TABLE 5:** Several state-of-the-art deep learning models for DR classification.

DL Systems	Algorithm	Training data set	Test data set	AUC	Sensitivity	Specificity	Aim of detection
Abràmoff et al. (10)	AlexNet/VGGNet	Messidor-2	10 primary care practice sites from the United States	NA	87	91	mtmDR
Gulshan et al. (17)	Inception-V3	EyePACS, Messidor-2	Messidor-2	0.99	87	99	referable DR, operating cut point with high specificity
					96	94	referable DR, operating cut point with high sensitivity
			EyePACS-1	0.99	90	98	referable DR, operating cut point with high specificity
					98	93	referable DR, operating cut point with high sensitivity
Zhang et al. (27)	Inception-V3	SAMS, SPPH	SAMS, SPPH	0.98	98	98	referable DR
Gulshan et al. (20)	Inception-V4	EyePACS, Messidor-2	Aravind	0.96	90	92	referable DR
			Sankara	0.98	92	95	referable DR
Bellemo et al. (28)	VGGNet/ResNet	SiDRP 2010-2013	Zambia mobile screening	0.97	92	89	referable DR
				0.98	92	95	STDR
Sayres et al. (29)	Inception-V4	EyePACS, 3 eye hospitals of India	EyePACS2	0.88	92	95	referable DR

*DL = deep learning, mtmDR = more than mild DR (ETDRS level 35 or higher and/or DNE), NA = not available, SAMS = The Sichuan Academy of Medical Sciences, SiDRP = Singapore Integrated Diabetic Retinopathy Screening program, SPPH = Sichuan Provincial Peoples Hospital, STDR = severe nonproliferative DR or worse.*

This study had the following advantages. The first was the study design. We conducted a self-controlled trial to verify the diagnostic efficacy of the AI software, EyeWisdom V1. The gold standard was the diagnostic results of the ophthalmologists group, which consisted of three senior researchers and a senior PI. PI was arbitrated when the three researchers have different diagnoses. The second was the distribution of DR at different periods in the data set. Referred to foreign studies of similar products, such as the public databases Eyepacs-1 and Messidor-2, the distribution of these data sets in the five DR was extremely uneven with the negative data of more than 90%. However, in China, the majority of patients had positive DR (20). Both theory and experience showed that the data distribution, model, and training process would affect the classification ability of the model, and then affect the sensitivity, specificity, and AUC value (23). Therefore, to minimize the impact of this aspect, the data distribution of this clinical trial was mainly concentrated on the positive data, which accounted for more than 70%. In the pre-test, the positive image data also occupied a very high proportion (positive ratio, 70.90%), and the positive data in this study accounted for 68.14%, which referred to the data of the pre-test.

At the same time, the selection of DL network should consider model effect, network complexity, video memory size as well as training and testing efficiency. An algorithm with a good recognition effect, relatively low network complexity, and high training and testing efficiency should be selected. In 2014, Inception and VGGNet won the first and second prizes in the ImageNet competition. Inception had an effective local network topology without fully connected layers and reduced the space of the algorithm greatly. After that, Inception had been updated with improved architecture and better performance. In 2015, ResNet, which had at most 152 convolutional layers was introduced and adopted the concept of residual learning and identity mapping (21, 22). Although normal neural networks developed for natural image classification could be directly implemented for DR grading, some modifications were made in EyeWisdom V1 including loss function and input image size. First, different from natural image classification in which categories were equal and had no internal relations, the five-level DR grade showed a severity that had clear order. For example, the misclassification from no DR to mild NPDR and from no DR to PDR were totally different. Thus, we adopt Kappa Loss for model training instead of the commonly used Cross Entropy Loss. Second, the size of the input image was also an important parameter of the network structure. To simulate the magnification function in the actual application to view the details of the images, we choose the images with larger sizes. Increasing the size of the input image allowed more detailed features to enter the model. On the other hand, due to the limitation of the size of the machine cache, the larger the input image size was, the smaller the number of images that can be contained in a batch was, which would affect the effect of model training. Considering that different network architectures showed inconstant performances in different classification targets or databases, experiments were made to choose the better network for DR grading among these CNN networks that were recognized as having better performance. Detailed information of the experiment is shown in Supplementary Datasheet 1. Ultimately, we chose the Inception-V3 network framework with the 896*896 image size as the final network structure.

Different from most software that took any DR and referral DR for detection purposes, such as Gulshan et al., Romero-Aroca et al. (17, 29) the output of EyeWisdom V1 was the DR grading score. In patients with referred DR, the classification of DR was clearly given, which could provide a reasonable interpretation for ophthalmologists. Doctors can reasonably arrange medical resources according to the severity of DR classification so that all patients can receive timely treatment.

However, there were some limitations in our study. Compared with IDX-DR products with wide-angle stereo images and optical coherence tomography (OCT) as reference images, EyeWisdom V1 in our study made referral recommendations based on the single fundus images of the posterior pole. There was a possibility of mistake and failure to report in our products due to the omission of peripheral retinal lesions. In addition, our sample size was relatively small and a more rigorous gold standard should be explored and more research data should be included in future studies.

In conclusion, the AI grading software, EyeWisdom V1, could provide clear DR classification and referral recommendations according to the fundus images of the posterior pole, it has a good consistency with ophthalmologists. Through EyeWisdom V1, accurate and credible diagnostic solutions could be provided. At the same time, the diagnostic efficiency is also greatly improved.

## Data Availability Statement

The original contributions presented in the study are included in the article/Supplementary Material, further inquiries can be directed to the corresponding author/s.

## Ethics Statement

Written informed consent was obtained from the individual(s) for the publication of any potentially identifiable images or data included in this article.

## Author Contributions

W-FZ conceived this study and wrote the draft of the manuscript. X-YZ conceived this study and revised the manuscript. D-HL, Q-JW, and D-YD designed the study, analyzed the data, and revised the manuscript. L-HM and Y-LW assisted in the draft. Y-XC conducted and coordinated the whole process. All authors have read the final manuscript and reached an agreement.

## Conflict of Interest

D-HL, Q-JW, and D-YD were employed by Visionary Intelligence Ltd. The remaining authors declare that the research was conducted in the absence of any commercial or financial relationships that could be construed as a potential conflict of interest.

## Publisher’s Note

All claims expressed in this article are solely those of the authors and do not necessarily represent those of their affiliated organizations, or those of the publisher, the editors and the reviewers. Any product that may be evaluated in this article, or claim that may be made by its manufacturer, is not guaranteed or endorsed by the publisher.

## Abbreviations

AI: artificial intelligence
API: application programming interface
AUC: area under the curve
CI: confidence interval
CCM: corneal confocal microscopy
CNN: convolutional neural network
DL: deep learning
DR: diabetic retinopathy
DME: diabetic macular edema
EC: Ethics Committee
ICMJE: International Committee of Medical Journal Editors
ICF: informed consent form
NPV: negative predictive values
OCT: optical coherence tomography
PDR: proliferative diabetic retinopathy
PPV: positive predictive values
QC: quality control
ROC: receiver operating characteristic
SGD: Stochastic Gradient Descent
STDR: sight-threatening diabetic retinopathy.
