# Supplementary material for: The Validation of Deep Learning-Based Grading Model for Diabetic Retinopathy
Source: Front Med (Lausanne). 2022 May 16;9:839088. doi: 10.3389/fmed.2022.839088 (PMC9148973; doi:10.3389/fmed.2022.839088)
Supplement: Supplementary file 1 [file Data_Sheet_1.PDF]

Supplement 1.

Development of the Algorithm

Annotation of the dataset

Fundus images of diabetics from two Chinese public hospitals (Beijing Tongren Hospital and Henan Provincial People's Hospital of China) were collected. Data processing engineers screened out the images including the non-fundus images such as OCT and FAF, non-posterior pole images and non-image files, only retained the "posterior pole fundus images". The image quality of 31082 posterior fundus images in total was evaluated. (Image quality was classified as "excellent", which means all lesions can be graded, "good" if there are only 1-2 factors affecting image quality, "adequate quality" if there are 3-4 problems that affect image quality but all lesions grading is not affected, "insufficient for full interpretation" if one or more lesions cannot be graded, "insufficient for any interpretation" and "others" if some other quality factors interfered with grading.) 20503 images with acceptable quality ("excellent", "good", "adequate" and "insufficient for full interpretation") were marked by ophthalmologists according to the items in the 2003 International Clinical DR Classification System. Our annotation team consisted of 29 trained ophthalmologists and they achieved a consistency of 80% in DR grading test. Each image was annotated by at least three ophthalmologists and questionable images were arbitrated by the associate chief physicians or chief physicians.

Quality control (QC) module

Resnet-34 convolutional neural network was used to determine image quality, which was a recognized mature algorithm. The network took pre-processed fundus images as input and output quality scores. Image quality scores ranged from 1 to 6, with 1, 2, 3, 4 being interpreted as acceptable image quality and 5, 6 as unacceptable. We used model parameters pre-trained on the ISLVRC dataset, a subset of ImageNet, as initialization parameters and 31082 images from public hospitals for training (25082), validation (3000) and internal testing (3000). Considering that in actual use, the difference of images may come from the equipment, the parameter selection or operation of the technicians, thus we tested the influence of image disturbance and

acquisition equipment on the module performance.

DR classification module

In order to find out the best network for DR classification, we compared the different combinations of three mature network architectures, (VGG16, ResNet-50 and Inception-V3) and image size. A network architecture Inception-V3 (image size 896*896), that achieved the best balance between performance and efficiency was selected (Supplement Table 1 and Supplement Figure 1, the database for this comparison was based on the test set in DR classification module).

Supplement Table 1. The Performance Comparison of The Different Combinations of Network and Image Size for referral DR.

Network \ Image Size	448*448		672*672		896*896	
	AUC	Kappa	AUC	Kappa	AUC	Kappa
VGG16	0.935	0.742	0.94	0.769	0.938	0.748
ResNet50	0.935	0.734	0.939	0.757	0.936	0.748
Inception-V3	0.92	0.688	0.935	0.735	**0.951**	**0.811**

Supplement Figure 1. The ROC curves of different combinations of Network and Image Size for referral DR.

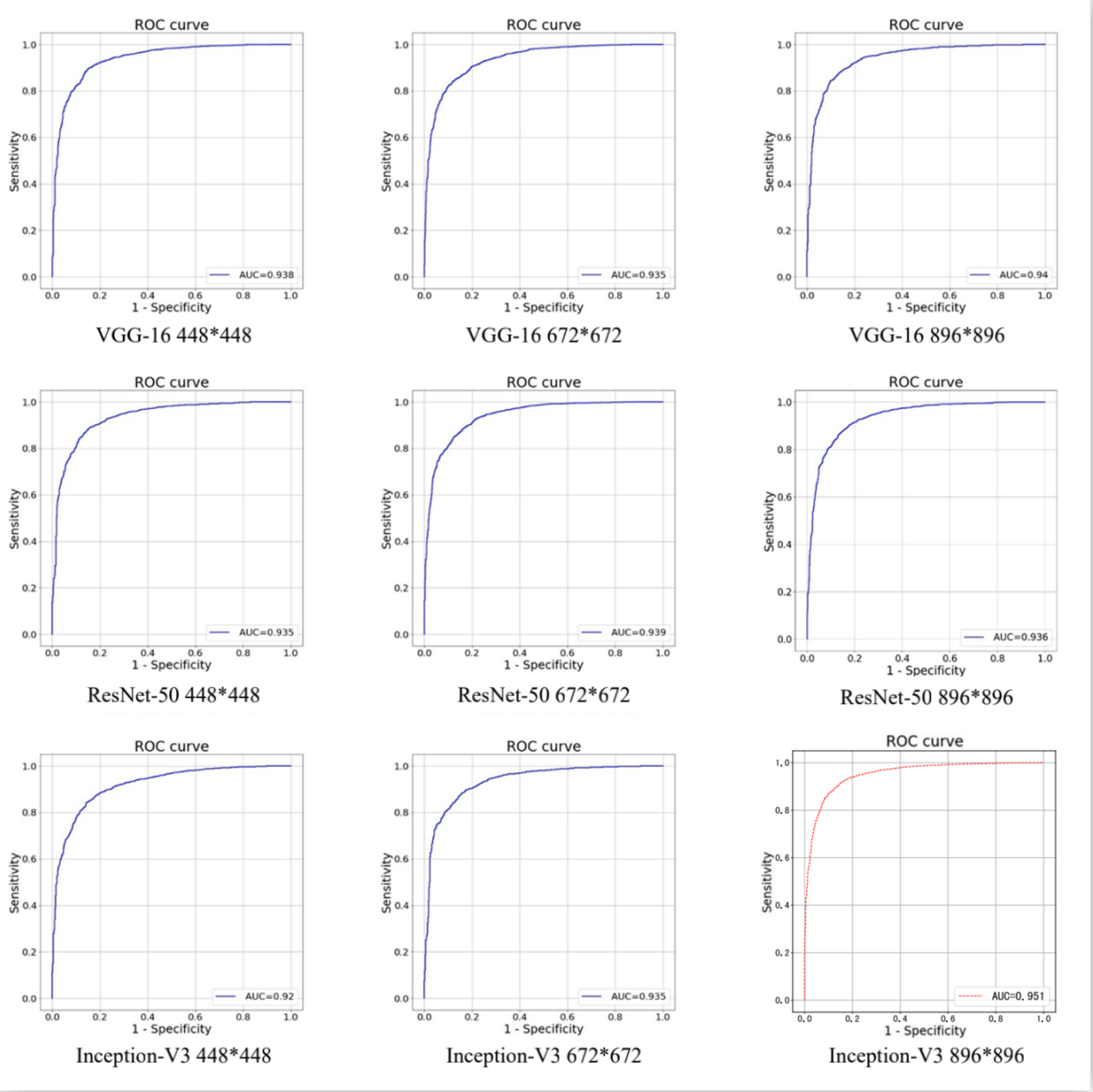

ROC=receiver operating characteristic, AUC=area under ROC curve.

The Inception-V3 network took 896*896 fundus images as input and output five scores at image level. There was no human-AI interaction in the handling of the input data. The five scores represented the network's certainty of no DR, mild NPDR, moderate NPDR, serve NPDR and PDR (or DR0 to DR4 for short), respectively. Training, validation and internal test set had 15611, 1640 and 3252 fundus images, respectively. The same as the QC module, we evaluated the performance of DR grading module under different image perturbations and cameras.

Performance of the AI Software in the internal test set

Quality control (QC) module

Training, validation and internal test set had 25082, 3000 and 3000 fundus images, respectively. The data distribution of different sets and image quality scores was shown in Supplement Table 2. The results showed that the sensitivity, specificity and the area under the receiver operating characteristic curve (AUC) of the algorithm were 92.9%, 92.4% and 0.980, respectively. When there was a disturbance on the fundus images that did not affect the image quality classification (such as the rotation, flipping and clipping), the change of statistical performance was less than 0.3% and the AUC value of the model measured under different camera brands fluctuated less than 2%.

Supplement Table 2. The Data Distribution of Different sets and Image Quality Scores for QC module.

**Category**	**Training set**	**Validation set**	**Test set**	**Total**
1(%)	767(3.06)	80(2.67)	97(3.23)	944
2(%)	5143(20.50)	618(20.60)	640(21.33)	6401
3(%)	8221(32.78)	968(32.27)	977(32.57)	10166
4(%)	4298(17.14)	507(16.90)	507(16.90)	**5312**
5(%)	72(0.29)	11(0.37)	14(0.47)	**97**
6(%)	6581(26.24)	816(27.20)	765(25.50)	8162
Total	25082	3000	3000	31082

DR classification module

Training, validation and internal test set had 15611, 1640 and 3252 fundus images, respectively. The data distribution of different sets and DR grading was shown in Supplement Table 3. The results demonstrated that the AUC, sensitivity and specificity of referable DR were 0.951, 90.2% and 86.1%, respectively. The change of statistical performance under different image perturbations was less than 0.2% and the AUC value of the model measured under different camera brands fluctuated less than

5%.

Supplement Table 3. The Data Distribution in Different sets for DR classification module.

Category	Training set	Validation set	Test set	Total
DR0(%)	1286(8.24)	155(9.45)	226(6.95)	1667
DR1(%)	2988(19.14)	280(17.07)	588(18.08)	3856
DR2(%)	7525(48.20)	787(47.99)	1565(48.12)	9877
DR3(%)	2072(13.27)	229(13.96)	464(14.27)	2765
DR4(%)	1740(11.15)	189(11.52)	409(12.58)	2338
Total	15611	1640	3252	20503
