# Supplementary material for: The Validation of Deep Learning-Based Grading Model for Diabetic Retinopathy
Source: Front Med (Lausanne). 2022 May 16;9:839088. doi: 10.3389/fmed.2022.839088 (PMC9148973; doi:10.3389/fmed.2022.839088)
Supplement: Supplementary file 2 [file Data_Sheet_2.PDF]

Supplement 2.

2.1. Pre-test 1. A preliminary test of expected value.

Before the formal trial, the diagnostic performance of EyeWisdom V1 was preliminarily evaluated on 227 fundus images provided by Peking Union Medical College Hospital. The results were shown in the following Supplement Table 4 and 5.

Supplement Table 4. Distribution of fundus images in 5-DR of Pre-test 1 (n=227).

Distribution	Number	Percentage (%)
DR0	32	14.1%
DR1	24	10.6%
DR2	88	38.8%
DR3	37	16.3%
DR4	46	20.3%
Total	227	100.0%

DR=diabetic retinopathy

Supplement Table 5. Distribution of DR grading of ophthalmologists and EyeWisdom V1.

Manual group / AI group	Positive (%)	Negative (%)	Total (%)
Positive (%)	161(70.9)	4(1.8)	165(72.7)
Negative (%)	10(4.4)	52(22.9)	62(27.3)
Total (%)	171(75.3)	56(24.7)	227(100.0)

DR=diabetic retinopathy, Negative=DR0+DR1, Positive=DR2+DR3+DR4

The results showed that the sensitivity, specificity and DAR of the EyeWisdom V1 in the pre-test were 94.2% (95%CI 89.5%-97.2%), 92.9% (95%CI 82.7%-98.0%) and 93.8% (95%CI 89.9%-96.6%), respectively. The positive data of this pre-test in the tertiary A-level hospital was very high (positive ratio, 70.90%). Since the sensitivity and specificity of diagnostic tests would be affected by the distribution of positive and negative samples, the data of the pre-test would be used as the reference basis for the clinical study. Thus, the sample distribution of pre-test and formal

clinical trial can be considered from the same whole.

2.2. Pre-test 2. A preliminary test of target value.

In this pre-test, 173 fundus photos of DR were randomly selected from the Peking Union Medical College Hospital and diagnosed by four attending ophthalmologists of two public hospitals (Peking Union Medical College Hospital, Eye Hospital China Academy of Chinese Medical Sciences) respectively to simulate the diagnostic ability of ophthalmologists in lower hospitals. The results were as follows.

Supplement Table 6. Distribution of fundus photos in 5-DR of Pre-test 2 (n=173).

Distribution	Number	Percentage (%)
DR0	6	4%
DR1	14	8%
DR2	78	45%
DR3	33	19%
DR4	42	24%
Total	173	100.0%

DR=diabetic retinopathy

Supplement Table 7. Distribution of DR grading of four attending ophthalmologists.

Ophthalmologists	Positive	Negative	Total
A	132	41	173
B	124	49	173
C	159	14	173
D	122	51	173

DR=diabetic retinopathy, Negative=DR0+DR1, Positive=DR2+DR3+DR4

The sensitivity of four attending ophthalmologists were 84.97%, 79.74%, 98.69% and 76.47%, respectively. The specificity of them were 90.00%, 90.00%, 60.00% and 75.00%, respectively. The mean sensitivity and specificity of them were 84.97% and 78.75%. The diagnostic capability of the lower hospital was more in line with the main application environment after the software was launched. Therefore, the target

value set in this study should not be lower than the level of the pre-test.
