# Supplementary material for: The Validation of Deep Learning-Based Grading Model for Diabetic Retinopathy
Source: Front Med (Lausanne). 2022 May 16;9:839088. doi: 10.3389/fmed.2022.839088 (PMC9148973; doi:10.3389/fmed.2022.839088)
Supplement: Supplementary file 3 [file Data_Sheet_3.PDF]

Supplement 3. The DAR and Kappa among graders for detecting any DR and referable DR.

Category	Any DR detection(95% CI)		Referable DR detection(95% CI)	
	DAR	Kappa	DAR	Kappa
**Grader A vs B**	94.73% (93.22% ~ 95.98%)	0.888 (0.860 ~ 0.916)	95.00% (93.53% ~ 96.23%)	0.898 (0.872 ~ 0.925)
**Grader B vs C**	90.47% (88.56% ~ 92.16%)	0.789 (0.750 ~ 0.828)	91.12% (89.26% ~ 92.75%)	0.817 (0.782 ~ 0.852)
**Grader A vs C**	89.64% (87.67% ~ 91.39%)	0.773 (0.733 ~ 0.813)	91.67% (89.87% ~ 93.25%)	0.828 (0.794 ~ 0.862)

DAR=diagnostic accordance rate, DR=diabetic retinopathy
